# Priority interventions to improve maternal and child diets in Sub‐Saharan Africa and South Asia

**DOI:** 10.1111/mcn.12526

**Published:** 2017-10-03

**Authors:** William A. Masters, Katherine Rosettie, Sarah Kranz, Sarah H. Pedersen, Patrick Webb, Goodarz Danaei, Dariush Mozaffarian, on behalf of the Global Nutrition and Policy Consortium, Olayinka Adekugbe, Ramesh Kant Adhikar, Archana Amatya, Gudina Egata Atomsa, Jane Badham, Lalita Bhattacharjee, Manav Bhattarai, Kaleab Baye, Mesfin Beyero, Viral Brahmbhatt, S. Chandrasekhar, Ram Krishna Chandyo, Cheryl Christensen, Namukolo Covic, Babukiika Dalton, Sonalde Desai, Charlotte Dufour, Patrizia Fracassi, Zewditu Getahun, Seema Gulati, Jemal Haidar, Tesfaye Hailu, Umesh Kapil, Nabeeha Kazi‐Hutchins, Aweke Kebede, Joyce Kinabo, Jamal Bakari Kussaga, Carol Levin, George Mavrotas, Ranju Mehta, Sailesh Mohan, Wilson Waiswa Mwanja, Babatunde Oguntona, Abiodun Oladipo, Ruth Oniang'o, Robert Paarlberg, Pooja Pandey Rana, D. Prabhakaran, V. Prakash, Seema Puri, S. K. Roy, Rekha Sharma, Sabnam Shivakoti, Simbarashe Sibanda, Roger Sodjinou, Andrew Thorne‐Lyman, Carol Tom, Geeta Trilok‐Kumar, Steven Vosti, Henry Wamani, Akwilina Wendelin

**Affiliations:** ^1^ Friedman School of Nutrition Science and Policy Tufts University Medford Massachusetts USA; ^2^ T.H. Chan School of Public Health Harvard University Cambridge Massachusetts USA

**Keywords:** diet quality, food systems, malnutrition, nutrition‐sensitive agriculture, priority setting, programme design

## Abstract

Nutrition‐sensitive interventions to improve overall diet quality are increasingly needed to improve maternal and child health. This study demonstrates feasibility of a structured process to leverage local expertise in formulating programmes tailored for current circumstances in South Asia and Africa. We assembled 41 stakeholders in 2 regional workshops and followed a prespecified protocol to elicit programme designs listing the human and other resources required, the intervention's mechanism for impact on diets, target foods and nutrients, target populations, and contact information for partners needed to implement the desired programme. Via this protocol, participants described 48 distinct interventions, which we then compared against international recommendations and global goals. Local stakeholders' priorities focused on postharvest food systems to improve access to nutrient‐dense products (75% of the 48 programmes) and on production of animal sourced foods (58%), as well as education and social marketing (23%) and direct transfers to meet food needs (12.5%). Each programme included an average of 3.2 distinct elements aligned with those recommended by United Nations system agencies in the Framework for Action produced by the Second International Conference on Nutrition in 2014 and the Compendium of Actions for Nutrition developed for the *Renewed Efforts Against Child Hunger* initiative in 2016. Our results demonstrate that a participatory process can help local experts identify their own priorities for future investments, as a first step in a novel process of rigorous, transparent, and independent priority setting to improve diets among those at greatest risk of undernutrition.

**Abbreviations used**
ICN2: Second International Conference on Nutrition
SA: South Asia
SSA: Sub‐Saharan Africa

## 1 INTRODUCTION AND MOTIVATION

Suboptimal diets are among the world's leading causes of death and disability (Black et al., 2008; Steiber et al., 2015), including through the contribution of low diet quality to poor maternal and child health outcomes (Allen, 2013). Associations between suboptimal diet and maternal and child health outcomes are especially important in Sub‐Saharan Africa (SSA) and South Asia, where current diets contribute to widespread stunting, wasting, and intrauterine growth restriction as well as micronutrient deficiencies in women and children (Black et al., 2008).

Governments around the world now acknowledge that improving diet quality is of increasing importance (United Nations, 2016a; United Nations Children's Fund, World Health Organization, & World Bank, 2015) and have defined nutrition‐related targets such as those outlined in the Sustainable Development Goals (SDGs; United Nations, 2016b), the Nutrition for Growth Summit in 2016 (“Nutrition for Growth Summit”, 2016), and the Second International Conference on Nutrition (ICN2) in 2014 (Food and Agriculture Organization & World Health Organization, 2014). The United Nations (UN) declared 2016 the start of a “Decade of Action on Nutrition” (the Decade) to accelerate progress in achieving global nutrition targets. The Decade calls for global, national, and regional stakeholders to take action on nutrition commitments outlined by the ICN2 and SDGs (Food and Agriculture Organization & World Health Organization, 2016).

To achieve global development goals, an important next step is to build consensus on which programmes and policies should be prioritized. Previous studies have compared interventions that deliver specific nutrients through supplementation or fortification (Bhutta et al., 2008; Fiedler & Puett, 2015; Shekar, Dayton Eberwein, & Kakietek, 2016), but the task of improving diets—which may be more effective in the long term—calls for a more diverse and complex set of mechanisms (Burlingame & Dernini, 2011; Jacobs & Tapsell, 2007). As a result, the 2013 Lancet Series on maternal and child nutrition specifically called for the “development of methods to allow comparison and evaluation of complex programmes with many objectives and joint outcomes” (Ruel et al., 2013).

This study describes the first steps in a novel approach towards systematic comparative effectiveness and cost‐effectiveness analysis of interventions to improve diets, using mixed methods to identify a high‐priority set of programmes and policies for further analysis and potential implementation in SSA and South Asia—the regions of the world with the least contemporary progress and largest remaining burdens of malnutrition (Black et al., 2008). Identifying the most promising programmes and policies for scale‐up could help decision makers optimize resource use and achieve nutrition goals as efficiently as possible, achieving the very large total economic and social gains available from well‐designed interventions (Alderman, Behrman, & Puett, 2017).

**Key messages**

- Interventions targeting diverse foods and risk factors are needed to improve maternal and child nutrition.
- Setting priorities for dietary interventions requires a wide range of expertise, methods, and data.
- We use a novel participatory approach involving 41 local experts to identify a set of 48 priority interventions for eight countries in South Asia and Africa.
- Priorities defined through regional consultations align with recommendations and goals of international organizations, with emphasis on combining multiple interventions to improve diet quality and target transfers of food, vouchers, or other resources to at‐risk mothers and children.

## 2 METHODS

The methods we use to identify high‐priority dietary interventions combine best practices in evidence synthesis and participatory research (Hill, Gonzalez, & Pelletier, 2011; Holdsworth et al., 2015; Sharma et al., 2017), while offering a novel approach to comparative effectiveness and cost‐effectiveness analysis. The consultative methodology is designed around the data needed to measure the health consequences of dietary changes, based on input from independent experts convened in global and regional advisory groups to provide a wide range of expertise and overcome concerns about lack of transparency and potential bias in previous cost‐effectiveness studies. The prespecified sequence we followed was designed and implemented with support from the Bill & Melinda Gates Foundation over a 4‐year period ending in 2017.

To identify the most promising nutrition‐sensitive strategies for improved maternal‐child health in SSA and South Asia, we followed a four‐step procedure: (a) identification of the most relevant diet–disease targets for maternal‐child health in these regions, based on qualitative expert assessments to design systematic reviews and quantitative meta‐analyses of the diet–disease relationships with strongest evidence; (b) identifying categories and characteristics of potential interventions, based on consultation with global experts and qualitative reviews; (c) prioritization and formulation of specific programmes, based on participatory consultation and deliberation with multidisciplinary regional and national experts from diverse sectors; and (d) validation of those results by comparison with lists made by international organizations. The resulting set of specific interventions presented in this paper will also be used in future studies for cost‐effectiveness analysis, by validating cost estimates against budgets for other activities and measuring effectiveness based on epidemiological models of risk reduction.

### 2.1 Identification of relevant diet–disease targets

To select relevant diet–disease targets, we performed consultations with experts to solicit recommendations regarding diet–disease relationships having high‐quality evidence on the following: (a) reliable data on current intake levels and etiologic effects, (b) high magnitude of current disease burden in South Asia and SSA, and (c) ability to intervene with one or more dietary interventions (Table S1). This process identified five pairs of dietary risk factors and associated diseases: iron and anaemia, zinc and stunting, zinc and diarrhoea, animal protein and stunting, vitamin A and mortality, and omega‐3 fatty acids and neurodevelopment. Details on criteria for selection of diet–disease pairs are provided in Table S1. Examples of other diet–disease pairs that were considered and not incorporated were iodine and neurodevelopment (omitted due to dominance of fortification strategies over dietary ones) and diet diversity and underweight (omitted due to insufficient evidence on effect size).

For each of the identified diet–disease pairs of interest, we separately summarized the available data for etiologic effects, including heterogeneity in these effects based on underlying characteristics of the intervention or targeted population, based on systematic reviews and meta‐analyses. Some of the preliminary results of these quantitative meta‐analyses have been reported (Pimpin et al., 2016; Pimpin, Kranz, Fawzi, Duggan, & Mozaffarian, 2016; Shulkin et al., 2016); the final analyses are ongoing and will be reported separately.

### 2.2 Categories and characteristics of nutrition‐sensitive programmes

To describe a set of potential interventions for comparative effectiveness and cost‐effectiveness analysis, we convened a cost‐effectiveness advisory group (CEAG) and performed a qualitative review of existing nutrition‐sensitive programmes focusing on maternal and child health. The CEAG is composed of independent researchers and technical experts with a diversity of expertise regarding cost‐effectiveness analysis for maternal and child health (Table S2). We focused on eight priority nations, based on their high burdens of malnutrition and maternal‐child disease as well as Gates Foundation nutrition priorities, including three in South Asia (India, Nepal, and Bangladesh) and five in SSA (Ghana, Tanzania, Uganda, Nigeria, and Ethiopia).

Through consultation with the CEAG and review of existing programmes, we identified six domains of nutrition‐sensitive programmes of high relevance and promise, largely consistent with the foci of the Lancet series paper on nutrition‐sensitive actions (Ruel et al., 2013), including (a) targeted and conditional transfers; (b) mass media and community‐based education; (c) preschool and school‐based feeding; (d) small‐scale and household production; (e) food processing and fortification; and (f) food markets and prices. The mechanisms of impact that these nutrition‐sensitive programmes utilize to alter dietary intake include the following: (a) transfer of resources to alter the purchase or use of home‐grown foods (hereafter referred to as resource transfers); (b) changing food prices and transaction costs to alter purchasing behaviour (hereafter referred to as access changes); (c) changing dietary preferences to alter the purchase or use of foods with existing household resources (hereafter referred to as preference changes); and (d) transfer of food items that are consumed without altering other choices (hereafter referred to as food transfers). We excluded programme domains whose mechanisms of impact were through more distal and indirect pathways, such as programmes focusing on economic development, women's empowerment, and the national supply of foods through agricultural production and international trade. We also excluded nutrition‐specific programmes that introduce particular nutrients through supplements rather than foods, but we did invite participants to consider including interventions that would scale‐up use of existing nutrient‐dense products such as biofortified crops or improved infant foods. Research and development efforts to formulate and produce these foods, as well as nutrition‐specific programmes such as breastfeeding promotion or supplementation with particular nutrients, are outside the scope of the present investigation. This ensured that our process would identify all available nutrition‐sensitive approaches to dietary change.

### 2.3 Prioritization and formulation of specific interventions

To characterize and formulate a set of specific programmes for prioritization for the eight countries of interest, we incorporated the diverse perspectives and expertise of regional experts through two separate meetings: one for South Asia, held in Kathmandu on December 8–10, 2015; and one for SSA, held in Addis Ababa on February 24–26, 2016. These meetings aimed to engage a diverse and representative set of stakeholders from civil society, private, academic, and nongovernmental organization sectors, including multilateral and bilateral organizations, specializing in the areas of nutrition, health, agriculture, policy, and economics. Potential participants were identified by the investigators, the CEAG, Gates Foundation contacts, and other expert contacts, based on their policy experience, scientific contributions, or positions in decision‐making bodies in the food system. Details about the attendees and the organizations represented are provided in Table S3.

Using standard principles of participatory research (Hill et al., 2011; Holdsworth et al., 2015), we sought active engagement through transparency and openness to diverse points of view. In‐depth discussion was facilitated by alternating between plenary group discussion periods and small breakout groups comprising four to six regional experts coupled with a facilitator and a recorder. Each intervention domain was introduced in plenary session, after which each breakout group then further developed and formulated a specific priority intervention in that domain.

The groups were instructed to formulate a nutrition‐sensitive intervention of their choice based on the following criteria: (a) interest in comparative effectiveness and cost‐effectiveness; (b) potential impact on dietary intake, from current in‐country experiences and other available evidence; (c) logistical and practical feasibility, as evidenced by implementation in similar contexts or other expert knowledge; and (d) likelihood of improving health disparities or meeting another specific population need. A fifth criterion, (e) legal and political feasibility, was also considered, with the caveat that highly promising interventions that met the other criteria but seemed currently legally or politically infeasible could be included, as the findings from the present process might improve their legal or political feasibility. Meetings utilized Chatham House Rules (i.e., all information would be recorded and utilized, but without specific attribution to neither the identity nor affiliation of any specific participant) to allow free and frank discussion of all available evidence.

For each priority programme, breakout groups described the following: (a) resources (human, financial, and inputs) required for intervention implementation; (b) mechanism for impact on diet; (c) target foods and/or nutrients to be increased; (d) location and demographic characteristics of the target population; (e) the lead authority and implementing organization for the intervention; (f) start‐up and recurring resources and the activities costs associated with implementation, maintenance, and evaluation; and (g) additional expert contacts relevant to the intervention. Following the meeting, documentation of each proposed programme was compiled, standardized, and shared with the regional experts and, if relevant, additional expert contacts to verify accuracy, clarify any discrepancies, and maintain transparency and accountability.

### 2.4 Validation of results against international benchmarks

National governments and international organizations use many different forums to share evidence and coordinate their actions, most notably through the UN system. The most recent and thematically closest benchmark for validating the interventions addressed in this study is the set of nutrition‐sensitive priorities for agriculture and food systems published in late 2016 as the Compendium of Actions on Nutrition (CAN) by the UN Network for Scaling Up Nutrition (Renewed Efforts Against Child Hunger and Undernutrition [REACH], 2016). For context, we also compare our results to the broader and earlier set of recommendations made at the ICN2 convened by UN agencies in November 2014 (Food and Agriculture Organization & World Health Organization, 2014) and the SDGs adopted by UN member states in late 2015 (United Nations, 2016b).

REACH is a partnership between the major UN system agencies involved in maternal and child nutrition, namely, the Food and Agriculture Organization, the United Nations Children's Fund, the World Food Programme, the World Health Organization, and the International Fund for Agricultural Development. The CAN was developed through a participatory process that involved interagency discussions and exchanges with subject area experts. The goal of REACH is to aid national governments in the scale‐up of nutrition actions and promote official support at the highest levels of international cooperation. The CAN identifies nine actions that fall into five categories (livestock and fisheries; crops/horticulture; food processing, fortification, and storage; food consumption practices for healthy diets; and enabling environment) that fall within the scope of this project (Table 1). Other potential actions in the REACH framework that fall outside the scope of this project are interventions in maternal and childcare, health systems, and nutrition governance.

**Table 1:** Priority programme interventions identified by our participatory approach as compared to REACH compendium of actions for nutrition^a^

REACH category and action	Identified priority programmes
Livestock and fisheries	
Animal husbandry, fisheries, and insect farming	• Backyard poultry production in Nepal
	• Household animal and horticulture production in Ghana
	• Aquaculture development in Uganda
	• Aquaculture development and nutrition education in Uganda
	• School livestock programme in Ethiopia
	• Conditional livestock transfer in Ethiopia
	• Targeted cash and chicken transfer in Uganda
Crops/horticulture	
Diversification and locally adapted varieties	• Home gardens in India
	• Kitchen gardens in Bangladesh
	• School‐based fruit production in Tanzania
	• Home gardens and small livestock production in Uganda
	• Home gardens and small livestock production in Bangladesh
Food processing, fortification, and storage	
Food processing (excl. fortification)	• Complementary food processing programme in Ghana
Fortification (including salt iodization and fortification of complementary foods)	• Point of consumption fortification in Nepal
	• Micronutrient sachets for home fortification in India
	• Home‐based fortified flour production in Nepal
	• Complementary food production in Ethiopia
	• Mass media campaign on fortification with dried fish in Nigeria
Food consumption practices for healthy diets	
Food‐based nutrition education	• School‐based feeding and nutrition education in Ethiopia
	• Adolescent health and nutrition education in South Asia
	• School‐based nutrition education in Ghana
	• School‐based nutrition education in Uganda
	• School‐based agriculture education in Ghana
Creating supportive environments to promote healthy diets in different settings	• School snack programme in Nepal
	• Preschool feeding in Bangladesh
Enabling environment	
Fiscal policy	• Milk transport subsidy in Nepal (livestock and fisheries)
	• Decreasing transport costs in Tanzania (food consumption)
	• Rice subsidy in Nigeria (food processing, fortification, and storage)
	• Food tax and subsidy in Ghana (food consumption)
	• Solar drier subsidy in Nepal (food processing, fortification, and storage)
Legislation, regulations/standards, protocols, and guidelines	• Food marketing association in Ghana (food consumption)
	• Improved traditional wet markets in Tanzania (food consumption)
	• Local market development in Nepal (food consumption)
	• Quality assurance for infant complementary food (food consumption)
	• Government quality certification seal (food consumption)
	• Integrating nutrition into agriculture and health services in Tanzania (crops/horticulture)
Social norms: Education/sensitization, behaviour change communication, and social marketing	• Technology‐enabled behaviour change communication in Nigeria (food consumption)
	• Nutrition education and media campaign in India (food consumption)
	• Diet diversity media campaign in India (food consumption)
	• Maternal nutrition media campaign in India (food consumption)
	• Media and education campaign in Ethiopia (food consumption)
	• Edutainment in Ethiopia (food consumption)
Vouchers or transfers (not defined by REACH)	
	• Targeted and conditional cash transfer in India
	• Conditional cash transfer in Nigeria
	• Conditional cash and food transfers in Bangladesh
	• Food vouchers in India
	• Food vouchers in Tanzania
	• Conditional food transfer in Ethiopia

a Renewed Efforts Against Child Hunger and Undernutrition. “Food, agriculture & healthy diets: Compendium of actions for nutrition” 2012. Accessed at: http://www.reachpartnership.org/compendium-of-actions-for-nutrition

Beyond validation against the REACH framework, we also compare our results to the broader recommendations about all aspects of human nutrition identified at the ICN2 in November 2014 (Food and Agriculture Organization & World Health Organization, 2014). The ICN2 was an intergovernmental meeting with representatives from government, civil society, and the private sector—all of which engaged in plenary discussions focused on nutrition issues. The outcome of ICN2 was a set of 60 recommendations of which seven are in scope for this project, involving recommended actions for sustainable food systems promoting healthy diets, nutrition education, and social protection. Finally, we benchmark our results against targets set for the SDGs adopted by UN member states themselves in September 2015 (United Nations, 2016b). The SDGs are the broadest of all global frameworks, specifying 169 targets in pursuit of 17 goals, of which our project aims at the first two targets specified for Goal 2 to end hunger, achieve food security, and improve nutrition (United Nations, 2016b).

## 3 RESULTS

Our participatory process identified 48 nutrition‐sensitive interventions across the six intervention domains (Table S4). Our meeting location led to many programmes designed for delivery in Nepal or Ethiopia (*N* = 7 each); with somewhat fewer designed to be implemented in each of the other countries of interest (*N* = 4–6 for each country other than Ghana), the South Asia region overall (*N* = 4), or the SSA region overall (*N* = 6). Details on this geographic pattern are provided in Figure S1.

Our study results span programmes that are either currently being implemented are additions to existing programmes or are new interventions not currently being implemented but showing significant promise in the target countries. The range includes programmes that directly transfer food or resources, use media and education campaigns to change dietary preference, or alter the food environment to improve access to more healthful foods. To validate the participatory process results, we compare them to the international recommendations.

### 3.1 Comparison with UN system frameworks

The 48 interventions described at our regional expert meetings align closely with the UN system agencies' priorities for agriculture and food systems to promote healthy diets, as listed in Table 1. Many of the proposed programmes include actions in more than one of the five REACH categories, but are listed here by their primary objective. Of the 48 interventions identified, 7 aim primarily to raise more livestock and fish; 5 target production of staple crops and horticulture; 6 address food processing, fortification, and storage; 7 focus on food consumption practices; 17 involve creating and strengthening the enabling environment; and 6 act through social protection. This degree of alignment reveals a relatively strong interest among regional experts in activities other than farm production, focusing on how crop and livestock products are transformed and used in off‐farm food systems after harvest. It is particularly notable that one eighth (six of 48) of the interventions chosen at our regional meetings focused on cash or food safety net transfers.

The outcome of our participatory process can also be compared with recommendations of the broader ICN2 Framework for Action (Food and Agriculture Organization & World Health Organization, 2014). The ICN2 provided seven distinct recommendations for interventions within our project's scope. The interventions identified through our workshops often align with more than one recommendation, especially the 12 interventions that aim to both (a) strengthen local food production and processing and (b) promote dietary diversification. An additional 17 interventions aim to improve storage, preservation, transport, and distribution, whereas 5 interventions aim to implement nutrition education and information interventions; 6 aim to conduct appropriate social marketing campaigns; and 8 incorporate nutrition objectives into social protection, or use cash and food transfers to improve diets. Benchmarking against the ICN2 framework, again our participatory process is notable for its general alignment with UN priorities and focus on postharvest food systems and social protection.

A third validation approach was to compare the objectives of our 48 interventions with the development goals of UN member states (United Nations, 2016b). The scope of our priority setting process falls within SDG 2 “to end hunger and to end hunger, achieve food security and improved nutrition”, and particularly its first two targets: “2.1 By 2030, end hunger and ensure access by all people, in particular the poor and people in vulnerable situations, including infants, to safe, nutritious and sufficient food all year round” and “2.2 By 2030, end all forms of malnutrition, including achieving, by 2025, the internationally agreed targets on stunting and wasting in children under 5 years of age, and address the nutritional needs of adolescent girls, pregnant and lactating women and older persons.” All 48 of our interventions aim at both of these targets, by making existing food more nutritious and available to those at risk of undernutrition.

In summary, the 48 interventions identified through our participatory process align closely with the UN agencies' REACH, CAN, and ICN2 Framework for Action, in pursuit of targets 2.1 and 2.2 of the SDGs. Relative to these international benchmarks, it is notable that participants in our workshops placed a strong emphasis on postharvest food systems and targeted transfers and often chose interventions that cut across multiple sectors and categories. In other words, interventions developed by participatory means through our project aimed at the same objectives as UN agencies and member states, but often did so through systemic, cross‐cutting programmes that address several different kinds of REACH actions, ICN2 recommendations, or SDG targets. In addition, interventions that arose out of our workshops are unique in that they were whole food based, selected with specific nutrient targets in mind, and highlighted the importance of altering dietary patterns in achieving population‐level improvements in nutrition outcomes.

### 3.2 Targeted populations, mechanisms, and nutrients

For the purposes of this analysis, each programme element is defined in terms of one kind of change to one dietary risk factor in a particular population. Programmes are built of multiple elements, and to summarize the evidence base by which each element would alter diets, we create a matrix of target population, target nutrient, and the mechanism of impact (resource transfer, food transfer, preference change, and access change), as shown in Table 2. This matrix approach allows us to use a single framework in which to compare 48 programmes, each of which may employ multiple mechanisms to reach several targets. For example, a programme involving both children under five and also pregnant women would be categorized as having separate elements for each of these target populations. In our matrix, there are 152 programme elements. The largest number of programme elements targeted children under five (*N* = 45), followed by the general population (*N* = 42), and pregnant/lactating women (*N* = 36). On average, programmes had 3.2 elements per intervention.

**Table 2:** Characteristics of 48 identified priority programme interventions by target nutrient, target, population, and mechanism of dietary impact^a^

	Target nutrient^b^					
Target population and mechanism of impact^a^	Iron	Zinc	Vitamin A	Animal protein	Omega‐3 fatty acids	Total
Children under five	**10**	**10**	**11**	**9**	**5**	**45**
Resource transfer	0	0	1	1	0	2
Food transfer	8	9	8	6	4	35
Preference change	1	1	1	2	1	6
Access change	1	0	1	0	0	2
Schoolchildren/adolescents	**3**	**3**	**5**	**3**	**4**	**18**
Resource transfer	0	0	0	0	0	0
Food transfer	1	1	2	2	2	8
Preference change	2	2	3	1	2	10
Access change	0	0	0	0	0	0
Pregnant/lactating women	**8**	**6**	**10**	**8**	**4**	**36**
Resource transfer	3	1	3	1	0	8
Food transfer	3	4	5	5	3	20
Preference change	2	1	2	2	1	8
Access change	0	0	0	0	0	0
Reproductive age women	**3**	**2**	**3**	**2**	**1**	**11**
Resource transfer	0	0	0	0	0	0
Food transfer	1	1	1	1	1	5
Preference change	1	1	1	1	0	4
Access change	1	0	1	0	0	2
General population	**10**	**8**	**7**	**12**	**5**	**42**
Resource transfer	0	0	0	0	0	0
Food transfer	5	3	3	5	2	18
Preference change	1	1	1	1	0	4
Access change	4	4	3	6	3	20
Total	**34**	**29**	**36**	**34**	**19**	**152**

a Proposed programmes often targeted multiple populations and multiple nutrients.

b Target populations are specified in greater detail in Table S2.

Numbers in bold are subtotals or totals.

**Figure 1:**
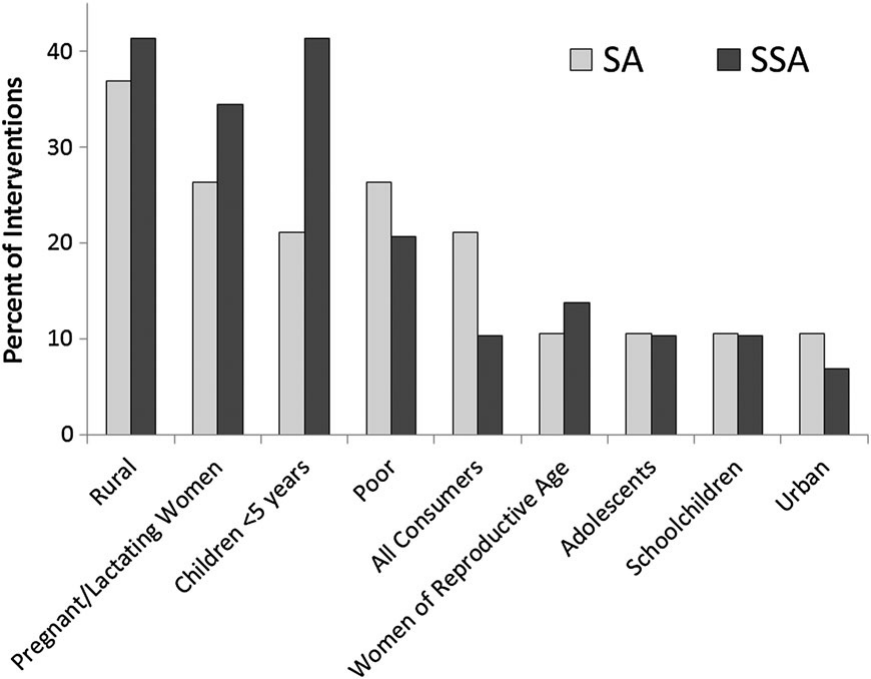
Percentage of priority programmes targeting each population category. Data shown are out of 48 interventions developed by participants at workshops in South Asia (SA) and Sub‐Saharan Africa (SSA). Because some programmes targeted more than one type of population, values may not sum to 100%

We identified both similarities and differences in the populations most often targeted in each region. In both South Asia and SSA, programmes targeting rural populations were most common. In contrast, pregnant and/or lactating women and especially children under 5 years were nearly equally targeted in SSA, but not in South Asia (Figure 1). In both regions, programmes targeting adolescents, school‐age children, and urban populations were the least commonly described. Further details on the target population(s) for each programme are provided in Table S4.

Of the four possible mechanisms for impact on dietary intake, food transfers were the most common accounting for a majority of all programme elements (*N* = 66; Table 2). The food transfer impact mechanism was particularly prominent among programme elements targeting children under five (*N* = 35) and pregnant/lactating women (*N* = 20). Programme elements involving resource transfers (i.e., cash transfers) were the least common (*N* = 10). Among programme elements targeting all consumers, impact pathways focused on changing environments and access (e.g., programmes aimed at food markets and prices) were most common (*N* = 20).

**Figure 2:**
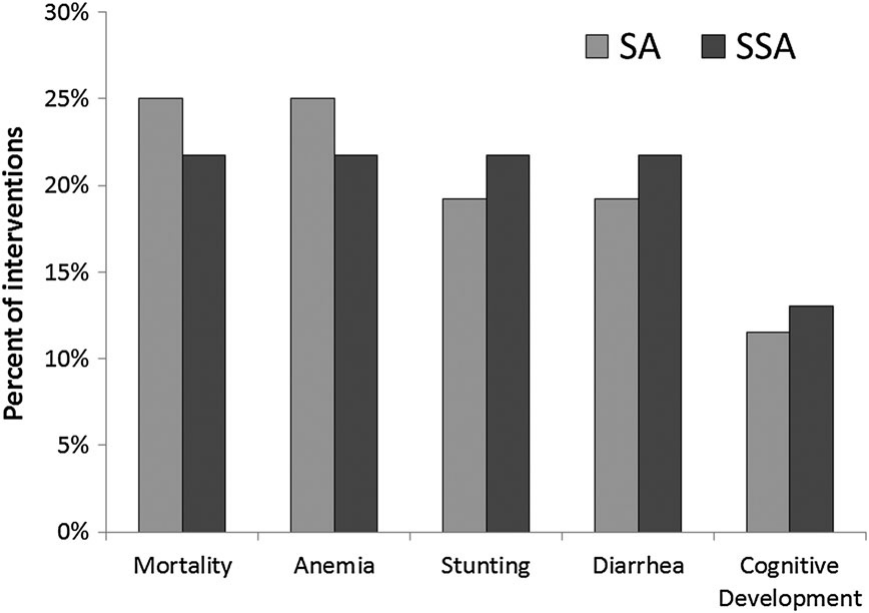
Percentage of priority programmes targeting each disease outcome. Data shown are out of 48 interventions developed by participants at workshops in South Asia (SA) and Sub‐Saharan Africa (SSA). Because some programmes targeted multiple disease outcomes, values may not sum to 100%

The programmes specified through our consultative process targeted distinct maternal and child health outcomes. Among the programmes proposed for South Asian countries, interventions aimed at child mortality and maternal/child anaemia were most common (25%; Figure 2). In contrast, for SSA, programmes equally prioritized child mortality (22%), child/maternal anaemia (22%), child stunting (22%), and child diarrhoea (22%). Programmes targeting childhood cognitive development were the least common in both regions (12% in South Asia; 13% in SSA).

**Figure 3:**
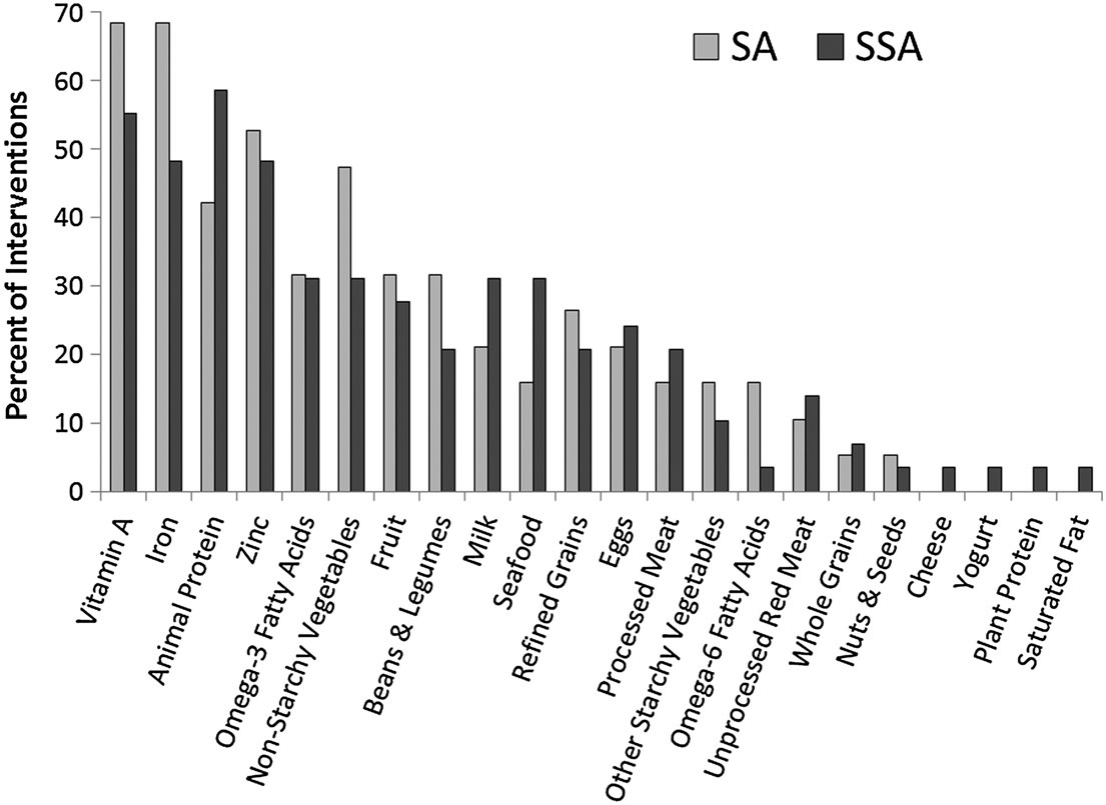
Percentage of priority programmes targeting each dietary component. Data shown are out of 48 interventions developed by participants at workshops in South Asia (SA) and Sub‐Saharan Africa (SSA). Because some programmes targeted more than one food or nutrient, values may not sum to 100%

Among individual dietary and nutrient factors, dietary vitamin A and iron were most commonly targeted in South Asia; whereas in SSA, vitamin A, iron, animal protein, and zinc were all relatively equally targeted (Figure 3). Among different foods, nonstarchy vegetables were the most common priority target for programmes in both regions, whereas programmes aiming to increase consumption of fruit, beans, and legumes were comparatively more common in South Asia, and programmes aiming to increase seafood and milk were comparatively more common in SSA. More details on target foods and nutrients for each proposed programme are provided in Table S4.

When assessing the programme elements across the 48 priority programmes, dietary vitamin A was the most commonly targeted (*N* = 36 programme elements), followed by dietary iron (*N* = 34), and zinc (*N* = 34; Table 2). For programme elements targeting children under five, most targeted vitamin A (*N* = 11), iron (*N* = 10), and zinc (*N* = 10). For programme elements targeting pregnant and/or lactating women, the majority targeted vitamin A (*N* = 10), animal protein (*N* = 8), and iron (*N* = 8). Programme elements targeting the general population largely focused on increasing dietary intakes of iron (*N* = 10) and animal protein (*N* = 12).

## 4 DISCUSSION

The results presented here represent a set of nutrition‐sensitive policy and programme priorities defined by national and international stakeholders in SA and SSA. These programmes were selected based on evidence for an interest in their comparative effectiveness and cost‐effectiveness, logistical and practical feasibility, likelihood of improving health disparities, or another specific population needs, as well as their legal and political feasibility. These findings offer the “on the ground” priorities from a rigorous and novel, transparent and participatory approach to formulating interventions for improved diet quality and reduced maternal and child undernutrition in SA and SSA. In addition, because some of the countries had already reached a stage of scaling up proven interventions that were discussed at these regional meetings, their expertise facilitated the discussions with actual on the ground outcomes and results.

Our methodology produced a set of 48 interventions identified by expert stakeholders from diverse institutions across government, academic, nutrition, health, agriculture, policy, and economic sectors. Although population, dietary, and mechanistic targets were wide‐ranging, two main themes emerged.

A first key finding from results reported in Table 1 is that most interventions identified (36 of 48) targeted by local stakeholders concern postharvest aspects of the food system, aiming to improve crop diversification and raising of livestock breeds, and delivery and utilization in the diet, reflecting accumulated evidence in the international literature (e.g., Ruel et al., 2013) and the views of local researchers and decision makers (Holdsworth et al., 2015). Over 80% of these identified programmes use (30 of 36) address food markets and aim to help households acquire and use more nutritious food, whereas the remainder transfer resources directly to people at risk of malnutrition. Among the dozen interventions to help households grow more food, most (seven of 12) aimed primarily at animal‐sourced rather than purely plant‐based foods. This finding clearly reflects widespread recognition acceptance of the need for improvements beyond households' own production of nutrient‐dense crops, in postharvest and off‐farm systems that improve the food environment but also provide direct transfers where needed.

A second key finding from results in Table 2 is that our participatory process led to cross‐cutting interventions involving multiple components related to target population, target nutrients, and mechanism of impact (an average of 3.2, from 152 elements in 48 programmes). In addition, regional stakeholders identified priorities that cut across the multisectoral themes and recommendations presented as part of the UN agencies' REACH actions (REACH, 2016) as well as the ICN2 recommendations (Food and Agriculture Organization & World Health Organization, 2014). From our analysis of individual programme elements and validation with the REACH and ICN2 documents, the stakeholders' 48 priority interventions are based on a strong evidence base. Our project's bottom‐up aggregation of stakeholder experiences led to novel combinations of these elements whose potential synergies have yet to be explored, such as combining cash grants with in‐kind transfers and education. Future work in this project will use existing data on baseline risks and programme effect sizes to generate modelled estimates of impact and cost‐effectiveness, and other kinds of research will also be needed to identify interaction effects among interventions that may complement or substitute for each other in any given setting.

The results presented here are a first step towards more transparent, stakeholder involvement in prioritization of interventions for future comparative effectiveness and cost‐effectiveness analyses. After this bottom‐up identification of high‐priority programmes, the resources needed and costs incurred for each intervention must be validated, considering reach, scope, and scale. Evidence on effectiveness can be derived from existing experiences and analogous interventions. Quantitative models, such as country‐specific, discrete‐time microsimulation models, can then incorporate these inputs to estimate the cost‐effectiveness of the various priority interventions. Ultimately, such results will enable governments, nongovernmental organizations, and other institutions to prioritize the interventions in which they will invest to improve dietary habits and maternal‐child health in these regions.

Our investigation has several strengths. One is to avoid investigator bias in the evaluation of selected programmes, achieved through open consultation along a predefined research plan that helped identify priority programmes prior to estimating their costs and effects on both dietary intake and disease outcomes. Another strength is the inclusion of multisector stakeholders in the consultative process, to ensure that the results from our future comparative effectiveness and cost‐effectiveness analyses of nutrition interventions will generate evidence that is relevant to those who would be funding, implementing, and evaluating those interventions in the future. In addition, our approach is strong in that it takes into account region‐specific information about priorities and constraints, ensuring that programme characteristics are tailored to regional and national circumstances. Additionally, this process can be carried out relatively quickly once a group of stakeholders can be convened. A final strength of our approach is our focus on pairing dietary risk factors with disease outcomes in each of the 48 proposed interventions, thereby highlighting the importance of improving dietary quality in mitigating disease.

The principal limitation of our study involves the cost, duration, and extent of consultations involved in this novel effort. We conducted two regional workshops with 41 local experts. Each workshop involved 2 days of face‐to‐face discussion, with extensive preparation beforehand and validation afterwards by the research team and a nine‐member global CEAG. To our knowledge, this was the most intensive effort ever undertaken to involve local stakeholders in defining priority interventions for cost‐effectiveness analysis. This approach results in a trade‐off between expediency and less precise costing estimates. Results demonstrate the value of this effort, and future studies could either replicate our intensive approach or build on our experience to expand the participatory process with more local experts over a longer time. Such a project could use lower‐cost tools such as video conferencing and online collaboration to permit even more iteration and reflection, generating even more realistic and meaningful results than those presented here.

Another possible limitation of our approach was an under‐representation of current or recent government officials (Table S3). Although meeting attendees considered programmes from a wide range of other perspectives, political and administrative support for these programmes could be as important as cost‐effectiveness for their sustainability. In focusing on the technical expertise needed for location‐specific programme design, our protocol provides just one step in the longer process of developing programmes that go on to attract sustained funding and political support.

## 5 CONCLUSION

In summary, this study demonstrates that a rigorous and transparent participatory process can generate an actionable set of programme priorities for policymakers' consideration and for cost‐effectiveness analysis, revealing both similarities and differences between local stakeholder priorities and international recommendations. Our approach overcomes several important limitations of previous research, finding that stakeholders' priorities combine multiple elements to address postharvest food systems and provide transfers beyond what households grow themselves or are able to acquire. In addition, we focus on diet, diet quality, and specific food and related nutrient targets that have relevance for a wide range of disease reduction targets. Therefore, our methods can be applied to future work that extends beyond maternal and child health and focuses on dietary intake and chronic disease reduction targets. Our future work will quantify the cost‐effectiveness of the interventions we identified through this approach, and their potential to achieve the goals of international agencies, national governments, and most importantly, the many individuals at risk of undernutrition in across Asia and Africa.

## CONFLICTS OF INTEREST

The authors declare that they have no conflicts of interest.

## CONTRIBUTIONS

DM designed research and obtained funding; DM, WM, SK, SP, PW, and GD conducted research; WM, KR, SK, and SP analysed data; WM, KR, PW, and DM wrote the paper; WM had primary responsibility for final content. All authors read and approved the final manuscript.

## Supporting information

Table S1.Diet‐disease pairs of interest and strength of evidence for causality, magnitude of burden, and potential to intervene.
Table S2. Members of the Cost‐Effectiveness Advisory Group
Table S3. Affiliations of the expert stakeholders who participated in the regional policy meetings.
Table S4. Program descriptions and key elements from regional meetings in Ethiopia and Nepal.
Figure S1. Geographic distribution of high‐priority nutrition‐sensitive programs proposed at regional meetings. Numbers in parentheses after each country name indicate the number of programs proposed for that country. Programs proposed for Nepal, India, Bangladesh, and South Asia (general) were from the regional meeting for South Asia held in Nepal; programs proposed for Nigeria, Ethiopia, Tanzania, Uganda, and Sub‐Saharan Africa (general) were from the regional meeting for Sub‐Saharan Africa held in Ethiopia.

## APPENDIX A.

### A..1

Olayinka Adekugbe (Save the Children)
Ramesh Kant Adhikar (Kathmandu Medical College)
Archana Amatya (Tribhuvan University Teaching Hospital)
Gudina Egata Atomsa (Haramaya University)
Jane Badham (JB Consultancy)
Lalita Bhattacharjee (FAO)
Manav Bhattarai (World Bank Nepal)
Kaleab Baye (Addis‐Ababa University)
Mesfin Beyero (Independent Consultant)
Viral Brahmbhatt (Nestle)
S. Chandrasekhar (Indira Gandhi Institute of Development Research)
Ram Krishna Chandyo (Kathmandu Medical College, Siddhi Memorial Hospital)
Cheryl Christensen (ERS)
Namukolo Covic (IFPRI)
Babukiika Dalton (USAID)
Sonalde Desai (University of Maryland)
Charlotte Dufour (FAO)
Patrizia Fracassi (Scaling up Nutrition)
Zewditu Getahun (UNICEF)
Seema Gulati (Nutrition Research Group)
Jemal Haidar (Addis Ababa University)
Tesfaye Hailu (Ethiopian Public Health Initiative)
Umesh Kapil (All India Institute of Medical Sciences)
Nabeeha Kazi‐Hutchins (Humanitas Global)
Aweke Kebede (Federal Ministry of Health, Ethiopia)
Joyce Kinabo (Sokoine University of Agriculture)
Jamal Bakari Kussaga (Sokoine University of Agriculture)
Carol Levin (University of Washington)
George Mavrotas (IFPRI)
Ranju Mehta (Institute of Medicine)
Sailesh Mohan (Public Health Foundation of India)
Wilson Waiswa Mwanja (Eccelenzia Consorzio Research and Management Team)
Babatunde Oguntona (Federal University of Agriculture)
Abiodun Oladipo (Micronutrient Initiative)
Ruth Oniang'o (Rural Outreach Africa)
Robert Paarlberg (Harvard Kennedy School)
Pooja Pandey Rana (Helen Keller International)
D. Prabhakaran (Public Health Foundation of India, Centre for Chronic Disease Control)
V. Prakash (Council of Scientific and Industrial Research)
Seema Puri (University of Delhi)
S. K. Roy (Bangladesh Breastfeeding Foundation)
Rekha Sharma (All India Institute of Medical Sciences, Diabetes Foundation India)
Sabnam Shivakoti (Department of Agriculture, Ministry of Agricultural Development, Government of Nepal)
Simbarashe Sibanda (FANRPAN)
Roger Sodjinou (UNICEF)
Andrew Thorne‐Lyman (Johns Hopkins University)
Carol Tom (Independent Consultant)
Geeta Trilok‐Kumar (University of Delhi)
Steven Vosti (University of California, Davis)
Henry Wamani (Makerere University)
Akwilina Wendelin (Sokoine University of Agriculture)
