# Supplementary material for: Priority interventions to improve maternal and child diets in Sub‐Saharan Africa and South Asia
Source: Matern Child Nutr. 2017 Oct 3;14(2):e12526. doi: 10.1111/mcn.12526 (PMC5901374; doi:10.1111/mcn.12526)
Supplement: Supplementary file 1 — Table S1.Diet‐disease pairs of interest and strength of evidence for causality, magnitude of burden, and potential to intervene. Table S2. Members of the Cost‐Effectiveness Advisory Group Table S3. Affiliations of the expert stakeholders who participated in the regional policy meetings. Table S4. Program descriptions and key elements from regional meetings in Ethiopia and Nepal. Figure S1. Geographic distribution of high‐priority nutrition‐sensitive programs proposed at regional meetings. Numbers in parentheses after each country name indicate the number of programs proposed for that country. Programs proposed for Nepal, India, Bangladesh, and South Asia (general) were from the regional meeting for South Asia held in Nepal; programs proposed for Nigeria, Ethiopia, Tanzania, Uganda, and Sub‐Saharan Africa (general) were from the regional meeting for Sub‐Saharan Africa held in Ethiopia. [file MCN-14-e12526-s001.docx]

| **Table S1. Diet-disease pairs of interest and strength of evidence for causality, magnitude of burden, and potential to intervene.** | | | | |
| --- | --- | --- | --- | --- |
| **Diet-disease pair** | **Reliable data on current intake** | **Reliable evidence for effect size** | **Magnitude of disease burdens in SA and SSA** | **Potential ability to intervene with dietary interventions** |
| Iron and anemia | Available | Strong | Large | Large |
| Zinc and stunting | Available | Moderate | Moderate to large | Large |
| Zinc and diarrhea | Available | Strong | Moderate to large | Large |
| Animal protein and stunting | Available | Moderate | Large | Large |
| Vitamin A and mortality | Available | Strong | Large | Large |
| Omega-3 fatty acids and neurodevelopment | Available | Moderate | Large | Large |

**Priority Interventions to Improve Maternal and Child Diets**

**in Sub-Saharan Africa and South Asia**

**Supplementary Materials**

*Updated 20 March 2017*

| **Table S2. Members of the Cost-Effectiveness Advisory Group** | |
| --- | --- |
| **Member** | **Affiliation** |
| Lalita Bhattacharjee | FAO |
| S Chandrasekhar | Indira Gandhi Institute of Development Research |
| Cheryl Christensen | ERS |
| Sonalde Desai | University of Maryland |
| Nabeeha Kazi-Hutchins | Humanitas Global |
| Carol Levin | University of Washington |
| Robert Paarlberg | Harvard Kennedy School |
| Steven Vosti | University of California, Davis |

| **Table S3. Affiliations of the expert stakeholders who participated in the regional policy meetings.*** | |
| --- | --- |
| **Stakeholder Groups** | **Institutional Affiliation** |
| **Government ministries**  SA (n=1)  SSA (n=1) | 1. Ministry of Agriculture 2. Ministry of Health |
| **Universities and national research organizations**  SA (n=8)  SSA (n=9) | 1. Addis Ababa University 2. All India Institute of Medical Science 3. Center of Nutrition and Metabolic Research, India 4. Council of Scientific and Industrial Research, India 5. Ethiopian Public Health Institute 6. Federal University of Agriculture, Nigeria 7. Haramiya University, Ethiopia 8. Kathmandu Medical College 9. Makerere University, Uganda 10. Institute of Medicine, Tribhuvan University, Nepal 11. Sokoine University of Agriculture, Tanzania 12. University of Delhi |
| **Civil society, NGOs and the private sector**  SA (n=4)  SSA (n=9) | 1. Bangladesh Breastfeeding Foundation 2. ECRAM 3. FANRPAN 4. Helen Keller International 5. Independent Consultants 6. International Confederation of Dietetic Associations 7. Micronutrient Initiative 8. Nestle 9. Rural Outreach Africa 10. Save the Children 11. Bill and Melinda Gates Foundation |
| **Multilateral and bilateral organizations**  SA (n=2)  SSA (n=7) | 1. FAO 2. IFPRI 3. UNDP 4. UNICEF 5. USAID 6. World Bank 7. WorldFish |
| ***** Nine research team members and 15 regional experts participated in the meeting held in the South Asia meeting held in Kathmandu, Nepal; and 13 research team members and 26 regional experts in the SubSaharan Africa meeting held in Addis Ababa, Ethiopia. At the South Asia meeting, 6 regional experts were from India, 7 from Nepal, 1 from Bangladesh, and 1 had general regional expertise. At the SubSaharan Africa meeting, 9 regional experts were from Ethiopia, 4 from Nigeria, 3 from Tanzania, 3 from Uganda, and 7 had general regional expertise. | |

| **Table S4. Program descriptions and key elements from regional meetings in Ethiopia and Nepal.** | | | | | | |
| --- | --- | --- | --- | --- | --- | --- |
| **Mechanism of impact**^1^ | **Country** | **Brief Description** | **Lead authorities or implementers** | **Target population** | **Target foods and nutrients^2^** | **Rationale for selection** |
| **Resource transfers** |  |  |  |  |  |  |
| Targeted & Conditional Cash Transfer | India | Pregnant women below the poverty line would receive a cash transfer conditional on a bank deposit of the benefit and hospital birth, coupled nutrition education. | Women and Child Development Ministry; Ministry of Human Resource Development | National; pregnant women below poverty line | Green leafy vegetables, **iron**, **vitamin A** | Helps to ensure women have control over money in household; can enhance existing cash transfer program |
| Conditional Cash Transfer | Nigeria | Pregnant women would receive cash transfers based on ANC attendance by mother and a family member plus delivery at a health facility. | Ministry of Health | Pregnant women in rural areas | Dietary diversity, **iron** | Early attendance at ANC improves maternal nutritional status and birth outcomes |
| Targeted Cash & Chicken Transfer | Uganda | Food insecure lactating/pregnant women and children under-5 would receive cash transfers and chickens during the lean season, coupled with nutrition education. | Local government (social services); agriculture extension workers, community-based organizations | Pregnant or lactating women & children under-5 in rural areas of Western region | Fruits, vegetables, chicken, dietary diversity, **animal protein, vitamin A** | Strong evidence base |
| Conditional Cash & Food Transfers | Bangladesh | Pregnant women would receive cash and fortified rice, fortified oil, and soybeans conditional upon attending ANC. | Ministry of Women and Children Affairs; Ministry of Health; national NGOs | National; pregnant women | Fortified rice, fortified oil, soybeans, **zinc, iron, vitamin A** | Ensure food and nutrition security as outlined in the National Social Security Strategy (NSSS) |
| **Food transfers** |  |  |  |  |  |  |
| Food Vouchers | India | Pregnant women and children under-5 would receive a prepared meal on-site or food vouchers, coupled with nutrition education. | State health ministry and/or social welfare | Pregnant women and children under-5 in rural and urban areas of 1 district | Milk, eggs, pulses, green leafy vegetables, animal source foods, oil, **zinc, iron, animal protein, omega-3 fatty acids, vitamin A** | Potential for cost-effectiveness and likely to have good reach through ANC visits |
| Conditional Livestock Transfer | Ethiopia | Pregnant women in PSNP districts would receive a dairy cow (or equivalent value in cash, if unable to care for a cow) conditional upon attending ANC. | Ministry of Agriculture | National; pregnant women | Milk, butter, **zinc, animal protein, vitamin A** | Evidence for large impact, high cost-effectiveness, and sustainability |
| **Table S4. (continued)** | | | | | | |
| **Mechanism of impact**^1^ | **Country** | **Brief Description** | **Lead authorities or implementers** | **Target population** | **Target foods and nutrients^2^** | **Rationale for selection** |
| Food Vouchers | Tanzania | Pregnant/lactating women and children 6-14 months would receive vouchers for animal-based foods, fruits, and vegetables. | Tanzania Social Action Fund | Pregnant/lactating women and children 6-14 months | Chicken, eggs, fish, fruits, vegetables, dietary diversity, **zinc, iron, animal protein, omega-3 fatty acids, vitamin A** | Will help meet maternal and child nutrition milestones |
| Conditional Food Transfer | Ethiopia | Food insecure women who are pregnant/lactating women or who have children under-5 would receive chickens and staple foods, conditional upon men engaging in public works programs and women/children attending ANC/child vaccination and health check-ups. | Ministries of Agriculture and Health; United States Agency for International Development; NGOs; local health clinics | Food insecure pregnant/lactating women and malnourished children under-5 | Eggs, chicken, fortified vegetable oil, fortified wheat flour, fortified cereals, pulses, soybeans, lentils, dietary diversity, **animal protein, vitamin A** | High political priority; will help to reduce poverty and food insecurity |
| Complementary Food Processing Program | Ghana | A semi-industrial food fortification program would be established at the community level that would produce and sell cereal-based CF mixed with powdered pulses and dried animal-based foods, coupled with nutrition education. | Local NGOs partnered with private businesses | Children 6-24 months in low-income households | Cereals, pulses, soy, fish, chicken, lentils, cowpeas, dietary diversity, **zinc, iron, animal protein, omega-3 fatty acids, vitamin A** | High political priority; high cost-effectiveness; strong evidence base |
| Home Gardens & Small Livestock Production | Uganda | Poor rural households would receive seeds and small livestock, coupled with nutrition education and technical training. | Ministries of Agriculure and Health; NGOs | Mothers and children under-5 in rural areas in the bottom 30% of wealth | Chicken, eggs, fruits, vegetables, cows, goats, milk, yogurt, cheese, dietary diversity, **iron, animal protein, vitamin A** | Improves dietary diversity and animal protein consumption |
| **Table S4. (continued)** | | | | | | |
| **Mechanism of impact**^1^ | **Country** | **Brief Description** | **Lead authorities or implementers** | **Target population** | **Target foods and nutrients^2^** | **Rationale for selection** |
| Point of Consumption Fortification | Nepal | Micronutrient powders would be manufactured locally and distributed by the Food Corporation of Nepal, coupled with a media campaign to increase awareness among mothers. | Food Corporation of Nepal; Ministry of Health | Children 6-24 months in 26 remote western districts | Cereal flour (soy, rice), **zinc, iro**n | High prevalence of anemia in Nepal; delivery system is already in place for rice and could be utilized for sachets |
| Micronutrient Sachets | India | Poor households with children 6-24 months would receive locally produced monthly rations of micronutrient sachets, coupled with an education component on adding sachets to meals (curries, daals). | Ministry of Food | Children under-5 in poorest 50% of population | Curry, daal, **zinc, iron** | Not provided |
| Home-Based Fortified Flour Production | Nepal | Mothers of children 6-24 months would receive home-based education and recipe development for micronutrient fortified super flour production. | Ministry of Health | Children 6-24 months in rural areas | Flour, rice, corn soy blend, bulgar wheat, chickpeas, **zinc, iron, vitamin A** | Home-based education will reach individuals who may not receive this education otherwise; utilizes food that is readily available |
| Complementary Food Production | Ethiopia | Semi-rural and rural women with infants 6-24 months would receive education on how to wash, dry, mill, and fortify grains with micronutrient powder to produce complementary foods for their own use or to sell to the local population. | NGO | Women with children 6-24mo. in rural and semi-urban areas | Grains, maize, sorghum, teff, wheat, barley, pulses, chickpeas, broad beans, field peas, **zinc, iron, vitamin A** | High political priority; local CF production will help with income generation |
| Home Gardens & Small Livestock | Bangladesh | Home gardens and small animal husbandry would be established in rural areas among poor households, in addition to education on income generation. | Government agencies and NGOs | Poor households in rural areas | Yellow/orange vegetables and fruits, chicken, goat, beef, **iron, animal protein, vitamin A** | HKI model exists and has been successful in the region; national models being taken to scale; government and NGOs appear ready for public-private partnerships in the area |

| **Table S4. (continued)** | | | | | | |
| --- | --- | --- | --- | --- | --- | --- |
| **Mechanism of impact**^1^ | **Country** | **Brief Description** | **Lead authorities or implementers** | **Target population** | **Target foods and nutrients^2^** | **Rationale for selection** |
| Backyard Poultry Production | Nepal | Poor individuals with agriculture experience in rural areas would receive ducks in conjunction with fertilizer for rice paddies and education on income generation; program would include education on raising ducks and increasing the nutritional value of their eggs. | Ministry of Agriculture; local NGO | Poor individuals in rural areas with agricultural experience | Duck meat, duck eggs, dietary diversity, **zinc, iron, animal protein, omega-3 fatty acids, vitamin A** | Not provided |
| Home Gardens | India | Home gardens would be established to diversify the food supply and generate income at the household level for small and marginal farmers (particularly women) who have agricultural or homestead land by providing seeds, supplies, tools, and veterinary services; would be coupled with an education component. | Ministry of Agriculture, Fisheries, and Livestock; cooperatives; NGOs; private organizations (e.g. Apple Board, Dairy Board) | Women in rural and peri-urban areas | Yellow/orange vegetables, green leafy vegetables, moringa, fruit, fish, eggs, chicken, pork, goat, beef, dietary diversity, **zinc, iron, animal protein, omega-3 FA, vitamin A** | Will provide diversified food supply at household level, linked to nutrition education |
| Kitchen Gardens | Bangladesh | Kitchen gardens would be introduced to increase consumption of fruits and vegetables. | Helen Keller International | Rural households and communities | Spinach, fenugreek, cucumbers, bananas, moringa, papaya, **iron, vitamin A** | Previous experience with this type of program from the 1960s shows success |
| Household Animal & Horticulture Production | Ghana | Poor households with an able body and plot of land would receive seedlings, seeds, and chickens plus training on how to grow fruits/vegetables and raise chickens. | Not provided | Households in bottom 40% of wealth | Fruits, vegetables, chicken, sweet potato leaves, cabbage, tomatoes, spinach, amaranth, carrots, pumpkin leaves, dietary diversity, **zinc, animal protein** | Will help to create a diversified, long-term food supply to improve food security |

| **Table S4. (continued)** | | | | | | |
| --- | --- | --- | --- | --- | --- | --- |
| **Mechanism of impact**^1^ | **Country** | **Brief Description** | **Lead authorities or implementers** | **Target population** | **Target foods and nutrients^2^** | **Rationale for selection** |
| Aquaculture Development | Uganda | Communities in the rural Western region would be taught how to build fishponds and have them stocked with small, oily, native fish species; coupled with education on fish harvesting, consumption, food safety, food processing, and hygiene | Ministry of Agriculture and Fisheries, working with local governments in sub-counties | Individuals living in rural areas in the western region | Fish, dietary diversity, **zinc, iron, animal protein, omega-3 FA** | Cultivation of a specific food that addresses several common nutrient deficiencies |
| Aquaculture Development and Nutrition Education | Uganda | Communities would build fishponds and receive bamboo fish cages; coupled with marketing/processing component for income generation, and nutrition education | Government fisheries/aquaculture program | Pregnant women and children under 2 living in 5 counties in Uganda | Zinc, omega-3 FA | Strong evidence base |
| School-Based Fruit Production | Tanzania | Primary schoolchildren would receive mango tree seedlings to plant at their homes, while each school would receive a solar drier to preserve excess mangoes for consumption in the off-season. | Ministries of Agriculture and Education; Department of Food Processing and Preservation | National; primary school children | Mangoes, vegetables, dietary diversity, **vitamin A** | Provides vitamin A in the off-season |
| School Snack Program | Nepal | Primary schoolchildren at government schools would receive a package of peanuts mixed with rice flakes each day in school as a snack. | Ministry of Education | National; primary schoolchildren | Peanuts, rice flakes, **omega-3 fatty acids** | Able to implement without a mid-day meal structure already in place |
| Preschool Feeding | Bangladesh | Early childhood education centers would serve nutrient-rich meals daily. | Ministry of Health and Family Welfare | Children ages 2-5 in the 4 poorest districts | Rice, fish, eggs, daal, soybean oil, green leafy vegetables, carrots, milk, **zinc, animal protein, vitamin A** | Not provided |
| School Livestock Program | Ethiopia | Schools in rural and peri-urban areas would receive dairy cows to provide milk to students during the school day; excess milk would be sold in local markets. | Ministry of Education; Ministry of Agriculture | Schoolchildren in rural and peri-urban areas | Milk, bread, dietary diversity, **zinc, animal protein, omega-3 fatty acids, vitamin A** | Strong evidence base |
| **Table S4. (continued)** | | | | | | |
| **Mechanism of impact**^1^ | **Country** | **Brief Description** | **Lead authorities or implementers** | **Target population** | **Target foods and nutrients^2^** | **Rationale for selection** |
| School-Based Feeding & Nutrition Education | Ethiopia | Children in grades 1-8 in rural communities would receive a hot meal made from local foods and school gardens, coupled with nutrition education. | Ministry of Education in collaboration with Ministries of Health, Agriculture, and Finance; possible collaboration with World Food Program and private sector | Small farming households and primary schoolchildren in rural areas | Staple grains, legumes, milk, eggs, rice, dietary diversity, **iron, animal protein** | High political priority; strong evidence base; will help improve dietary diversity, foster beneficial dietary habits, and help improve micronutrient deficiencies |
| **Preference change** |  |  |  |  |  |  |
| Nutrition Education & Media Campaign | India | Would provide education for schoolchildren on nutrition composition information and cooking techniques. In health facilities, billboards, cookbooks, pamphlets, powerpoints, and videos will be used to disseminate nutrition messages. | NGO with support from Ministry of Human Development; | National; schoolchildren and mothers | Bananas, dietary diversity | Not provided |
| Dietary Diversity Media Campaign | India | A combined mass media radio campaign and community-based nutrition education program would consist of weekly radio drama programs and daily short jingles, as well as community cooking demonstrations. | Ministry of Women and Child Development | Pregnant/lactating women and adolescent girls in rural and urban areas in 1 district | Carrots, pumpkin, mango, dietary diversity, **vitamin A** | Not provided |
| Maternal Nutrition Media Campaign | India | A television program would use a local celebrity mother as the star to promote higher caloric intake among pregnant and lactating women. | Ministry of Health; Information Ministry and Private Industry | National; pregnant/lactating women | Dietary diversity, **iron** | High impact and reach; no current interventions exist with this approach |
| Media & Education Campaign | Ethiopia | A radio and education campaign would promote increased intake of animal and plant-based protein and meal frequency using radio messages. Nutrition education would be delivered through the training of religious leaders who would deliver these messages to their congregations. | Ministry of Health; Ministry of Information and Technology | Pregnant/lactating women and women of reproductive age in rural areas | Milk, meat, eggs, fish, plant-based protein, dietary diversity, **zinc, iron, vitamin A, animal protein** | Evidence for large total impact |
| **Table S4. (continued)** | | | | | | |
| **Mechanism of impact**^1^ | **Country** | **Brief Description** | **Lead authorities or implementers** | **Target population** | **Target foods and nutrients^2^** | **Rationale for selection** |
| Edutainment | Ethiopia | Peer-to-peer videos would deliver nutrition messages to communities; coupled with discussions on prenatal nutrition, adolescent nutrition, complementary feeding, hygiene, and livestock productivity and consumption. | Ministries of Health and Agriculture | Adolescent girls, pregnant/lactating women, caregivers of children under-5, reproductive age women, farmers | Dietary diversity | Delivery platform with national reach already exists |
| Technology-Enabled Behavior Change Communication | Nigeria | Agricultural extension agents and community health workers would be trained in the delivery of core nutrition messages, prioritizing dietary change through the use of home production or farm income. | Digital Green | National; all consumers | Vegetables, dietary diversity, **zinc, iron, animal protein, vitamin A** | Evidence for high cost-effectiveness and large total impact; will help strengthen link between agriculture and nutrition |
| Integrating Nutrition into Agriculture & Health Services | Tanzania | A campaign would introduce agricultural links and infrastructure as a way to tie in nutrition messages targeting nutrients available through agricultural products. | Ministry of Health; Ministry of Agriculture; Ministry of Communications Department of Local Development | Smallholder households in rural areas | Eggs, dietary diversity | High political priority and evidence for large total impact |
| Adolescent Health & Nutrition Education | South Asia (country not specified) | Children in secondary schools would be provided with nutrition education; coupled with bi-annual school health and nutrition check-ups. Students would also receive a take-home pamphlet on health and nutrition that includes a growth-monitoring chart. | Ministry of Education; Ministry of Health | National; adolescent children | Dietary diversity; **zinc, iron, vitamin A, omega-3 fatty acids** | Not provided |
| School-Based Nutrition Education | Ghana | Public primary schools in poor areas would educate teachers, staff, and local unemployed parents on nutrition and cooking. Meals would be served in schools using local produce, and would be coupled with nutrition education for students. | Ministry of Education; Ministry of Health; Ministry of Agriculture; Ministry of Social Development | Children in the bottom 40% of wealth | Dietary diversity, **zinc, iron, animal protein, omega-3 fatty acids, vitamin A** | Multi-component program that engages the community to increase sustainability; evidence for large total impact |

| **Table S4. (continued)** | | | | | | |
| --- | --- | --- | --- | --- | --- | --- |
| **Mechanism of impact**^1^ | **Country** | **Brief Description** | **Lead authorities or implementers** | **Target population** | **Target foods and nutrients^2^** | **Rationale for selection** |
| School-Based Nutrition Education | Uganda | Children in grades 5-7 in public schools would receive education on life cycle nutrition, cooking, and basic agricultural production. | Ministry of Education; Ministry of Agriculture | National; adolescent children | Dietary diversity | Evidence for high cost-effectiveness |
| School-Based Agriculture Education | Ghana | Students would work together to manage a school farm and livestock. | Ministry of Education; District Officers of Education; Rural Outreach Africa; Ministry of Agriculture | National; adolescent children | Goat, milk, soy, ugali, dietary diversity | Program already in place and evidence for large impact |
| Quality Assurance for Infant Complementary Food | Tanzania | Food safety standards and quality assurance for locally produced CF would be established. | Tanzania National Bureau of Standards; Ministries of Health and Agriculture | National; children 6-24 months | Milk, grains, **animal protein** | Will help address problem of microtoxins in CF |
| Government Quality Certification Seal | Uganda | A government-led quality certification program for local CF would be implemented, coupled with a nutrition education and national awareness campaign to convey that these foods are nutritious for children. | Ministry of Trade; National Bureau of Standards; Chamber of Commerce; NGOs; private sector | Children 6-24 months in rural areas | Dietary diversity, **zinc, iron, vitamin A** | Will meet need for affordable fortified packaged infant CF, and need for quality assurance of such products |
| Mass media campaign on fortification with dried fish | Nigeria | A government initiative would be implemented targeting smallholder farms to encourage development of methods for safe, low cost, scalable production of sardines for use in fortification; chosen products would be marketed in a mass media campaign. | National fisheries authority; local university | National; pregnant women and children under-5 | Fish, **animal protein, omega-3 FA** | High cost-effectiveness |
| **Access changes** |  |  |  |  |  |  |
| Solar Drier Subsidy | Nepal | Solar driers for perishable, nutrient-dense foods would be subsidized to reduce food waste and to increase year-round availability. This would be coupled with a social marketing campaign. | Ministry of Agricultural Development; Alternative Energy Promotion Center | National; all consumers | Carrots, green leafy vegetables, pumpkin, fish, **animal protein, omega-3 fatty acids, vitamin A** | Not provided |
| **Table S4. (continued)** | | | | | | |
| **Mechanism of impact**^1^ | **Country** | **Brief Description** | **Lead authorities or implementers** | **Target population** | **Target foods and nutrients^2^** | **Rationale for selection** |
| Local Food Market Development | Nepal | Financially sustainable, diversified, private local food markets would be established through private specialty crop/agriculture boards by marketing cooperatives. | Ministries of Food, Commerce, and Consumer Affairs; private specialty crop boards | National; undernourished individuals | Dairy, green leafy vegetables, oranges, **zinc, iron, animal protein, vitamin A** | Thought to be superior to relaxing border restrictions |
| Milk transport Subsidy | Nepal | A refrigerated van for milk transport on dairy products would be introduced to reduce milk prices and retain quality. | Ministry of Livestock | National; all consumers | Dairy, **animal protein** | Would lower cost of milk for consumers |
| Decreasing Transport Costs | Tanzania | A seasonal program would give small business owners a truck and training on the distribution of produce from rural to urban areas, accompanied by a sales hub for centralized purchasing to ensure that farmers’ produce is picked up and sold at regular intervals. | Ministry of Commerce | Poor individuals in urban and semi-urban areas | Fruits, vegetables, beans, fish, nuts, dietary diversity, **iron, zinc, animal protein, vitamin A** | Would lower distribution costs, which are a major obstacle to accessing healthy foods |
| Rice Subsidy | Nigeria | Local ofada rice would be subsidized by guaranteeing a minimum buying price for the rice and reducing the price for consumers. Increased taxes on imported rice would fund subsidies for local rice seeds and equipment. | Ministry of Agriculture partnering with international NGOs | Pregnant/lactating women and children 6-24 months | Ofada rice, dietary diversity | High cost-effectiveness |
| Food Marketing Association | Ghana | Food marketing associations would be created to improve efficiency of the value chain for nutrient-dense foods. | Ministry of Agriculture; Ministry of Trade and Industry; local governments; International Fund for Agricultural Development | All consumers in rural areas | Fish, eggs, dairy, fruits, vegetables, dietary diversity, **zinc, iron, animal protein, omega-3 fatty acids** | Would help to make nutrient-dense foods more affordable and available |

| **Table S4. (continued)** | | | | | | |
| --- | --- | --- | --- | --- | --- | --- |
| **Mechanism of impact**^1^ | **Country** | **Brief Description** | **Lead authorities or implementers** | **Target population** | **Target foods and nutrients^2^** | **Rationale for selection** |
| Improved Traditional Wet Markets | Tanzania | The Ministry of Industry and Agriculture would give grants to villages to upgrade markets and technology to increase sales of nutritious, perishable products through improvements of traditional wholesale markets. | Ministries of Industry and Agriculture; public works authorities | Low-income individuals in poor areas | Meat, fish, dairy, **zinc, iron, animal protein, omega-3 fatty acids** | Not provided |
| Food Tax & Subsidy | Ghana | A flat 10% tax on sugar sweetened beverages would fund subsidies on fruits and vegetables for mothers and children by reducing the price of fresh fruits and vegetables; coupled with education and advocacy component on accessing healthy foods. | Ministries of Agriculture, Trade, Health, Finance | National; mothers and children under-5 | Fruits, vegetables, dietary diversity, **iron, vitamin A** | High cost-effectiveness; large total impact; strong evidence base |
| Abbreviations: ANC, antenatal care; PSNP, Productive Safety Net Program; CF, complementary food; NGO, non-governmental organization | | | | | | |
| ^1^Policies with multiple mechanisms of impact are categorized under the mechanism that constitutes the main focus of the program. | | | | | | |
| ^2^Target nutrients are in bold text. | | | | | | |


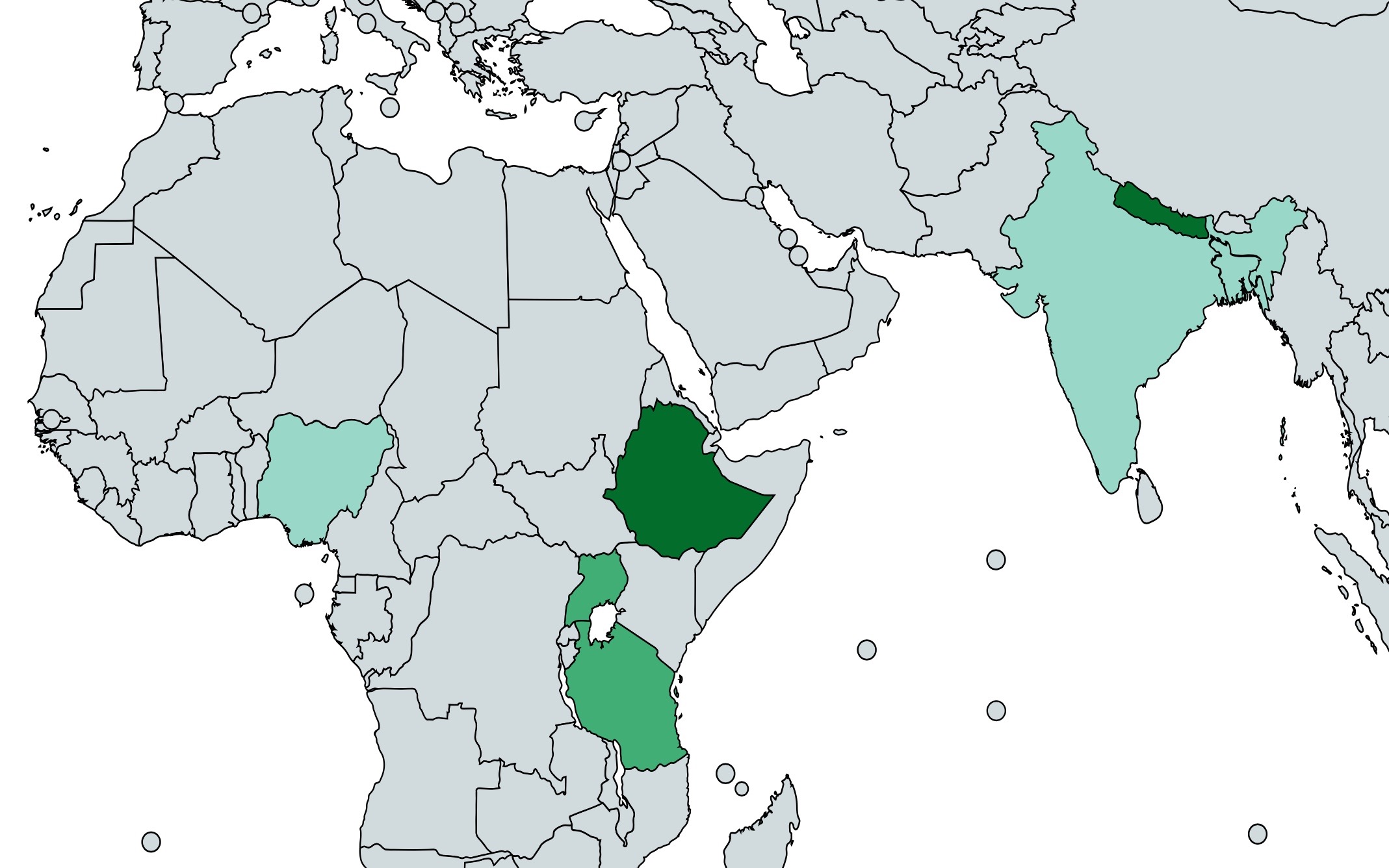


Bangladesh (4)

Nepal (7)

India (4)

South Asia (4)

Tanzania (6)

Uganda (6)

Ethiopia (7)

Nigeria (4)

Sub-Saharan Africa (6)

**Figure S1. Geographic distribution of high-priority nutrition-sensitive programs proposed at regional meetings.** Numbers in parentheses after each country name indicate the number of programs proposed for that country. Programs proposed for Nepal, India, Bangladesh, and South Asia (general) were from the regional meeting for South Asia held in Nepal; programs proposed for Nigeria, Ethiopia, Tanzania, Uganda, and Sub-Saharan Africa (general) were from the regional meeting for Sub-Saharan Africa held in Ethiopia.
